# Pantoprazole Attenuates MAPK (ERK1/2, JNK, p38)–NF-κB and Apoptosis Signaling Pathways after Renal Ischemia/Reperfusion Injury in Rats

**DOI:** 10.3390/ijms221910669

**Published:** 2021-10-01

**Authors:** Michael A. Fawzy, Sherif A. Maher, Sally M. Bakkar, Mahmoud A. El-Rehany, Moustafa Fathy

**Affiliations:** 1 Department of Biochemistry, Faculty of Pharmacy, Minia University, Minia 61519, Egypt; michael.fawzy777@mu.edu.eg; 2 Department of Biochemistry, Faculty of Pharmacy, Deraya University, Minia 61111, Egypt; sherif.ali@deraya.edu.eg (S.A.M.); mahmoud.elrehany@deraya.edu.eg (M.A.E.-R.); 3 Department of Biochemistry, Faculty of Medicine, Assiut University, Assiut 71515, Egypt; sallybakkar39@med.aun.edu.eg; 4 Department of Regenerative Medicine, Graduate School of Medicine and Pharmaceutical Sciences, University of Toyama, Toyama 930-0194, Japan

**Keywords:** pantoprazole, renal ischemia/reperfusion injury, reactive oxygen species, TNF-α, MAPK, NF-κB

## Abstract

Ischemia/reperfusion injury (IRI) in the kidney is the most common cause of acute renal dysfunction through different cell damage mechanisms. This study aimed to investigate, on molecular basics for the first time, the effect of pantoprazole on renal IRI in rats. Different biochemical parameters and oxidative stress markers were assessed. ELISA was used to estimate proinflammatory cytokines. qRT-PCR and western blot were used to investigate the gene and protein expression. Renal histopathological examination was also performed. IRI resulted in tissue damage, elevation of serum levels of creatinine, urea nitrogen, malondialdehyde, TNF-α, IL-6, IL-1β, up-regulation of NF-κB, JNK1/2, ERK1/2, p38, and cleaved caspase-3 proteins. Furthermore, it up-regulated the expression of the *Bax* gene and down-regulated the expression of the *Bcl*-2 gene. Treatment of the injured rats with pantoprazole, either single dose or multiple doses, significantly alleviated IRI-induced biochemical and histopathological changes, attenuated the levels of proinflammatory cytokines, down-regulated the expression of NF-κB, JNK1/2, ERK1/2, p38, and cleaved caspase-3 proteins, and the *Bax* gene, and up-regulated *Bcl*-2 gene expression. Moreover, treatment with pantoprazole multiple doses has an ameliorative effect that is greater than pantoprazole single-dose. In conclusion, pantoprazole diminished renal IRI via suppression of apoptosis, attenuation of the pro-inflammatory cytokines’ levels, and inhibition of the intracellular signaling pathway MAPK (ERK1/2, JNK, p38)–NF-κB.

## 1. Introduction

One of the underlying causes of acute renal failure is ischemia/reperfusion injury (IRI). It results from a sudden transient reduction in blood flow due to shock, trauma, abdominal surgery, arterial occlusion, or kidney transplantation, resulting in acute kidney injury (AKI). IRI is associated with tissue damage-impaired cell function. Several factors are involved in the pathophysiology of IRI, which may lead to organ injury and damage, such as the formation of free oxygen radicals, activation of inflammatory processes, mitochondrial damage, and finally chronic renal failure [1,2]. Kidneys are vulnerable to injury due to IRI, and this disorder can lead to worsening of renal dysfunction, leading to renal failure and renal-cell death [3].

Modulation of angiogenic, oxidative stress, and apoptotic pathways plays an important role in tissue disorders and restoration [4,5,6,7]. After renal IRI, primary phagocytic cells and macrophages play a crucial role in host defense and inflammation control [8]. Pro-inflammatory cytokines, mostly Tumor Necrosis Factor (TNF)-α, Interleukin-1 Beta (IL-1β) and Interleukin-6 (IL-6), are known for increasing reactive oxygen species (ROS)-production in renal cells. Thus, mitochondrial ROS are crucial for the full toxicity of cytokines [9]. Cytokines are essential mediators of immune response and inflammatory reactions and are involved in numerous biological processes [10,11,12,13]. Chronic renal failure is commonly presented with immune function abnormalities. These abnormalities are caused by impaired renal excretory function and uremic toxin accumulation in addition to necrosis and cell death [14,15].

Macrophages are activated by extracellular signaling pathways, including pro-inflammatory factors such as IL-1β, TNF-α, and IL-6, which in turn activate several intracellular signaling pathways, including the mammalian mitogen-activated protein kinase (MAPK) JNK/p38/ERK and the nuclear factor-kappaB (NF-κB) pathways [16,17]. To induce the expression of pro-inflammatory genes, NF-κB is activated firstly, then translocated into the nucleus, and is involved in a variety of pathological processes and inflammatory diseases [18,19]. The inhibition of the release of pro-inflammatory cytokines such as IL-1β, IL-6, and TNF-α is crucial for the control of AKI.

Repurposing drugs and looking for new therapeutic abilities of existed agents has received great attention [20,21,22,23,24]. Pantoprazole (PTZ) is an essential therapeutic agent for acid-related disorders. It is vital to triple therapy for the eradication of H.pylori. As monotherapy for healing of esophagitis and gastroesophageal reflux disease maintenance, PTZ has demonstrated comparable effectiveness to other proton-pump inhibitors and greater efficacy than histamine H2-antagonists. It was shown that it is also useful in avoiding rebleeding of ulcers, and it also has antioxidant activity by lowering calcium overload [25]. PTZ has a sulphoxide group that can scavenge hydroxyl radicals and protect cardiac cells through destruction of ROS by suppressing NADPH oxidase activity [26,27].

It was reported that, when administered 30 min before the induction of IRI, PTZ reduced malondialdehyde (MDA) level and toll-like receptor-4 protein expression in rats’ renal tissues [28]. This study aimed to investigate, for the first time, the possible molecular mechanisms underlying the effect of pantoprazole on renal IRI in rats by inspecting MAPK (ERK1/2, JNK, p38)–NF-κB and apoptosis signaling pathways.

## 2. Results

### 2.1. Effect of Pantoprazole on Different Biochemical Parameters

**Figure 1:**
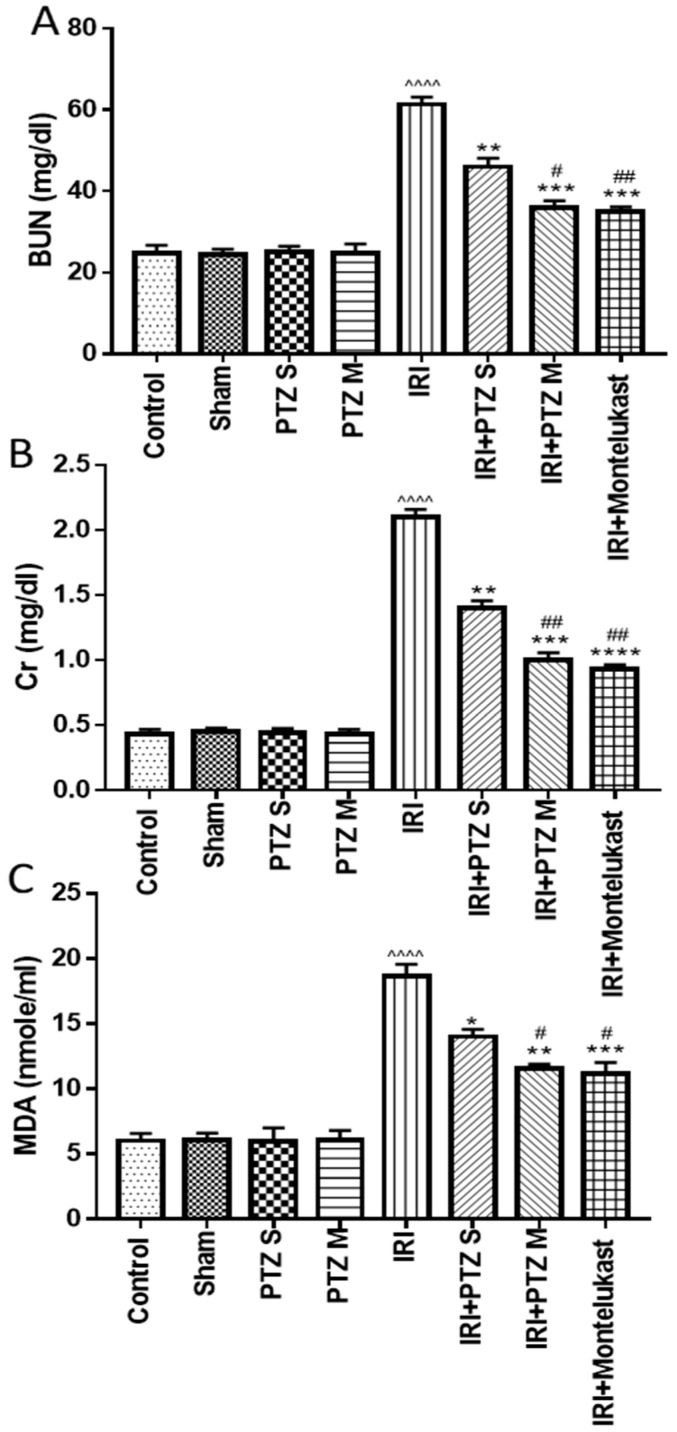
Effect of various treatments on serum levels of BUN (**A**), Scr (**B**), and MDA (**C**). Bars represent mean ± SEM. Significant difference between groups is analyzed by Student’s *t*-test after one-way ANOVA test (*n* = 8), where: ^^^^: *p* < 0.0001, compared to normal control group. *: *p* < 0.05, **: *p* < 0.01, ***: *p* < 0.001, and ****: *p* < 0.0001, compared to IRI group. #: *p* < 0.05, ##: *p* < 0.01, compared to (IRI + PTZ S) group.

To understand the effect of pantoprazole on the pathology of IRI, we analyzed treated rats for blood urea nitrogen (BUN) and serum creatinine (Scr) levels, both indicators of acute kidney injury. As shown in (Figure 1A,B), respectively, the biochemical analysis showed a significant (*p* < 0.0001) increase in BUN and Scr levels in IRI rats when compared to normal control rats. Treating IRI rats with pantoprazole single-dose (PTZ S) exerted a significant (*p* < 0.01, *p* < 0.05) decrease in BUN and Scr levels, respectively, compared to IRI rats. Furthermore, the BUN and Scr levels of IRI rats treated with pantoprazole multiple-dose (PTZ M) were significantly (*p* < 0.001) lower than those in IRI rats. Interestingly, treating IRI rats with PTZ M showed a significant (*p* < 0.05) reduction in BUN and Scr levels when compared to IRI rats treated with PTZ S.

Serum MDA is a marker for lipid peroxidation, oxidative stress, and tissue injury. Consistent with the alleviation of IRI-induced tissue damage by single- and multiple-dose pantoprazole treatment, we observed an increase in MDA levels in IRI rats which was dose-dependently affected by pantoprazole treatment (Figure 1C). Montelukast was used as a control treatment in these experiments which consistently showed protective effects (Figure 1A–C) as described previously [29].

### 2.2. Serum Levels of the Inflammatory Cytokines (TNF-α, IL-1β, and IL-6)

**Figure 2:**
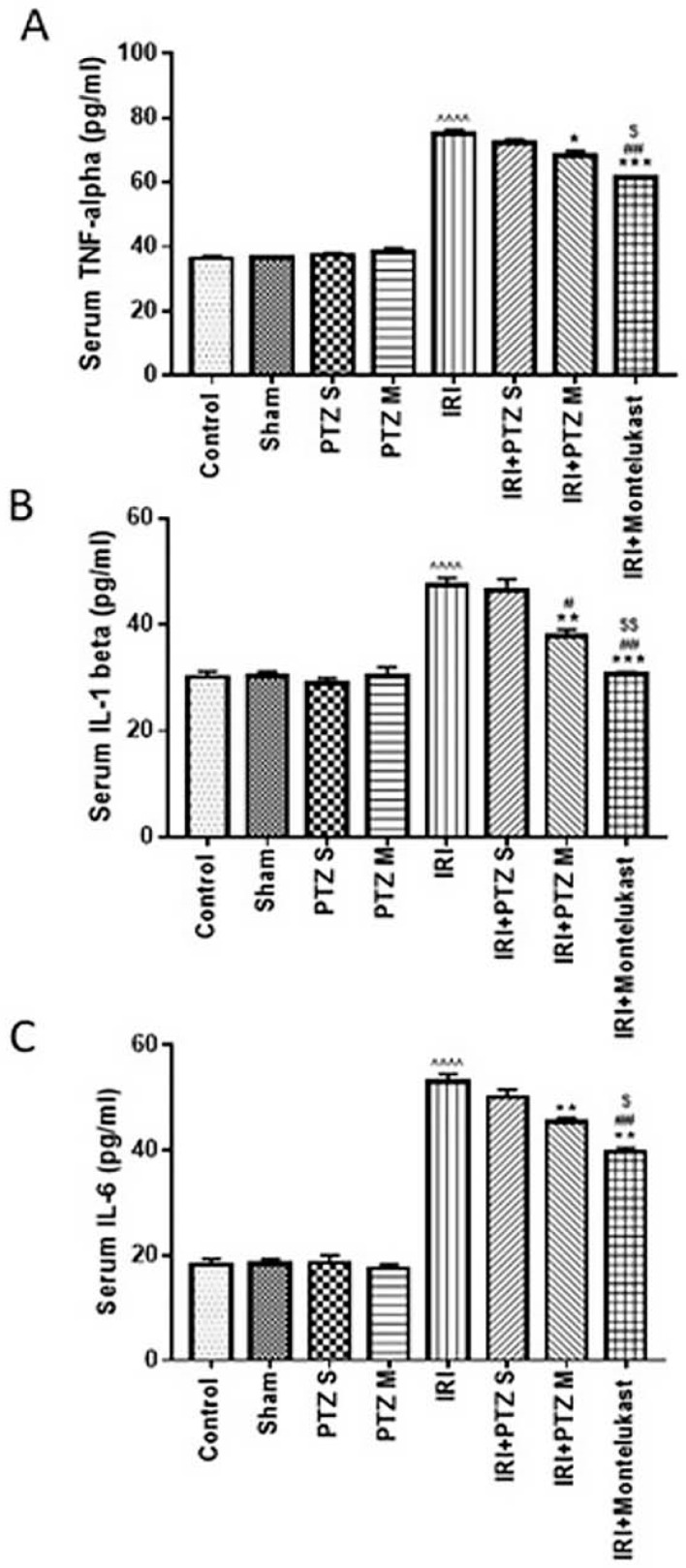
Effect on levels of inflammatory cytokines. Serum levels of (**A**) TNF-α, (**B**) IL-1β, and (**C**) IL-6 (pg/mL) for the different groups. Bars represent mean ± SEM. Significant difference between groups is analyzed by Student’s *t*-test after one-way ANOVA test (*n* = 8), where ^^^^: *p* < 0.0001, compared to normal control group. *: *p* < 0.05, **: *p* < 0.01, ***: *p* < 0.001, compared to IRI group. #: *p* < 0.05, ##: *p* < 0.01, compared to (IRI + PTZ S) group. $: *p* < 0.05, $$: *p* < 0.01, compared to (IRI + PTZ M) group.

To check the anti-inflammatory effect of pantoprazole on IRI rats, we investigated the treated rats for the pro-inflammatory mediators, TNF-α, IL-1β, and IL-6, in serum. As shown in (Figure 2), the measured pro-inflammatory cytokines TNF-α, IL-1β, and IL-6 in serum were significantly (*p* < 0.0001) increased in IRI rats when compared to normal control rats. Treating IRI rats with PTZ S showed no significant (*p* > 0.05) decrease in TNF-α, IL-1β, or IL-6 levels when compared to IRI rats. Meanwhile, treating IRI rats with PTZ M showed a significant (*p* < 0.05, *p* < 0.01, *p* < 0.01) decrease in TNF-α, IL-1β, and IL-6 levels, respectively, when compared to IRI rats. Furthermore, treating IRI rats with PTZ M showed a significant (*p* < 0.05) decrease in IL-1β levels when compared to treating IRI rats with PTZ S. Montelukast was, also, used as a control treatment in these experiments which exhibited protective effects.

### 2.3. Expression of B-Cell Lymphoma 2 (Bcl-2) and Bcl-2 Associated X-Protein (Bax) Genes

**Figure 3:**
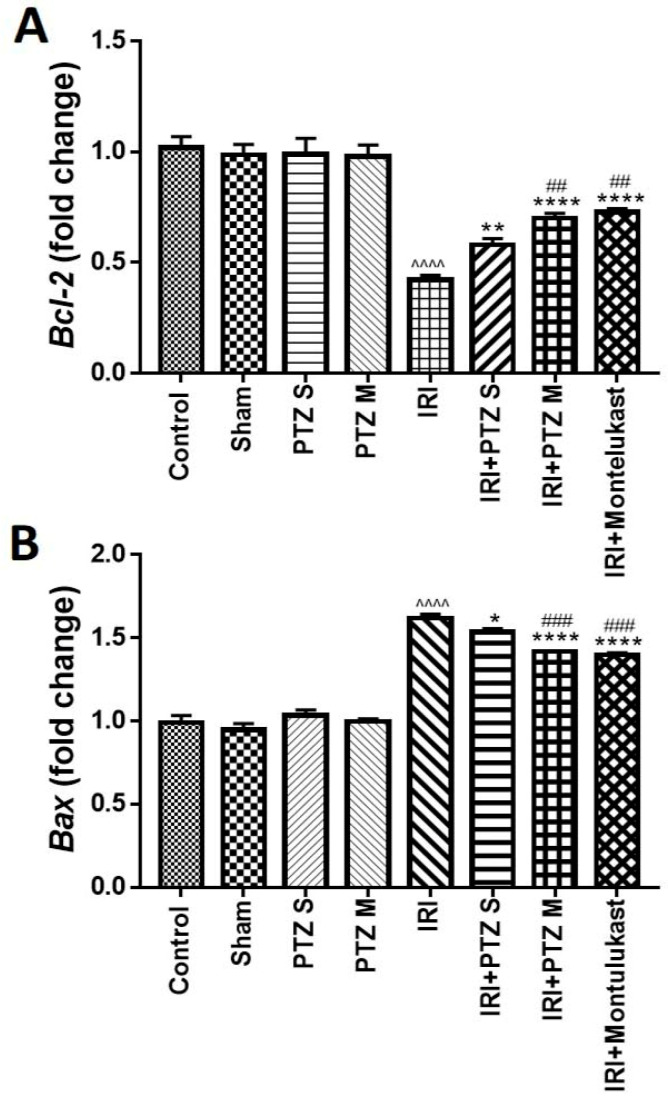
Expression of *Bcl-2* (**A**) *and Bax* (**B**) genes in renal tissues. Quantitative RT-PCR was used to evaluate the gene expression in renal tissues for rats of different groups. Data represent fold change relative to the normal control group expression after normalization to *GAPDH* gene. Bars represent mean ± SEM. Significant difference between groups is analyzed by Student’s *t*-test after one-way ANOVA test (*n* = 8), where: ^^^^: *p* < 0.0001, compared to normal control group. *: *p* < 0.05, **: *p* < 0.01, ****: *p* < 0.0001, compared to IRI group. ##: *p* < 0.01, ###: *p* < 0.001, compared to (IRI + PTZ S) group.

The expression of anti- and pro-apoptotic genes, *Bcl-2* and *Bax*, respectively, was investigated in the treated rats to evaluate the effect of pantoprazole on apoptosis. Relative to the expression of the normal control rats and after normalization to *Glyceraldehyde-3-phosphate dehydrogenase* (*GAPDH*) as a housekeeping gene, Figure 3A showed that IRI significantly (*p* < 0.0001) decreased renal mRNA levels of *Bcl-2*. Treating IRI rats with PTZ S and PTZ M exerted a significant (*p* < 0.01 and *p* < 0.0001, respectively) dose-dependent increase in *Bcl-2* gene expression when compared to IRI rats.

In addition, Figure 3B shows that IRI significantly (*p* < 0.0001) increased the renal mRNA levels of *Bax*, which is a pro-apoptotic gene, relative to rats of the normal control group. Furthermore, treating IRI rats with PTZ S and PTZ M showed a significant (*p* < 0.05 and *p* < 0.0001, respectively) decrease in renal mRNA levels of *Bax* when compared to IRI rats. Interestingly, treating IRI rats with PTZ M showed a significant (*p* < 0.001) reduction in renal mRNA levels of *Bax* when compared to IRI rats treated with PTZ S.

### 2.4. Expression of p-JNK1/2, p-ERK1/2, p-P38, Cleaved Caspase-3, and NF-κB Proteins

**Figure 4:**
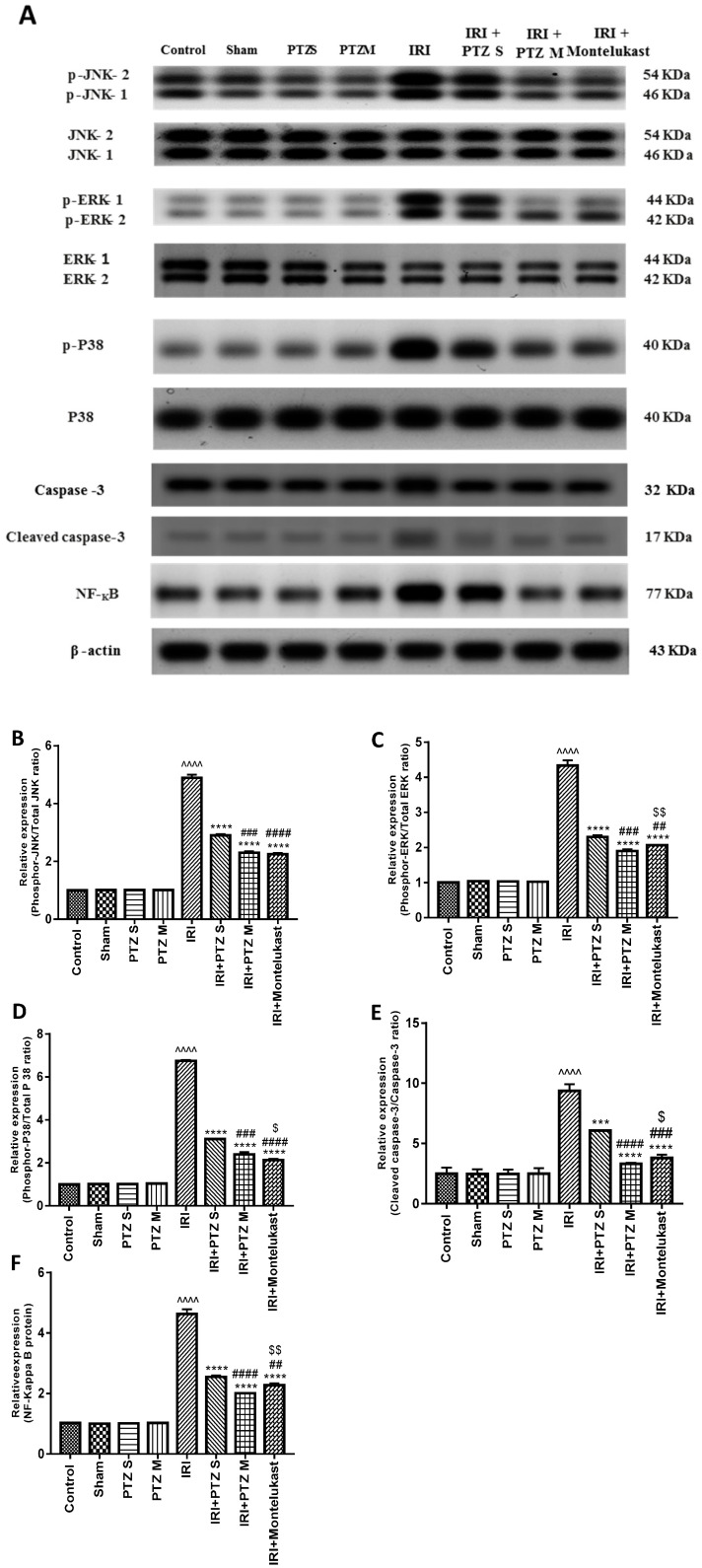
Effect of pantoprazole on the expression of JNK1/2, ERK1/2, p38, caspase-3, and NF-κB proteins. (**A**) Representative western blots of JNK 1/2, phosphor-JNK1/2, ERK1/2, phosphor-ERK1/2, p38, phosphor-p38, caspase-3, cleaved caspase-3, total NF-κB, and β-actin proteins for all groups. (**B**–**F**) Expressions of phosphor-JNK1/2/total JNK1/2, phosphor-ERK1/2/total ERK1/2, phosphor-P38/total P38, cleaved caspase-3/caspase-3 and total NF-κB proteins, respectively, were expressed densitometrically, using bands in (**A**) after normalization to the corresponding internal control β-actin, as fold change relative to that of normal control rats. Bars represent mean ± SEM. Significant difference between groups is analyzed by Student’s *t*-test after one-way ANOVA test, where: ^^^^: *p* < 0.0001, compared to normal control group. ***: *p* < 0.001, ****: *p* < 0.0001, compared to IRI group. ##: *p* < 0.01, ###: *p* < 0.001, ####: *p* < 0.0001, compared to (IRI + PTZ S) group. $: *p* < 0.05, $$: *p* < 0.01, compared to (IRI + PTZ M) group.

Western blotting was performed to check the influence of pantoprazole on various proteins’ expression in treated rats to explain the abovementioned obtained effects, using montelukast as a control treatment. Western blotting showed a significant (*p* < 0.0001) renal up-regulation of phosphor-JNK1/2/total JNK1/2, phosphor-ERK1/2/total ERK1/2, phosphor-P38/total P38, cleaved caspase-3/caspase-3, and total NF-κB proteins in injured rats compared to normal rats after normalizing the intensities of bands to β-actin. Interestingly, treating IRI rats with PTZ resulted in significant (*p* < 0.0001) down-regulation of all proteins compared to IRI rats. Furthermore, the expression level of all proteins in the (IRI + PTZ M) group was significantly lower than that in (IRI + PTZ S) group, as shown in (Figure 4).

### 2.5. Histopathological Examination for the Renal Tissues of Different Groups

**Figure 5:**
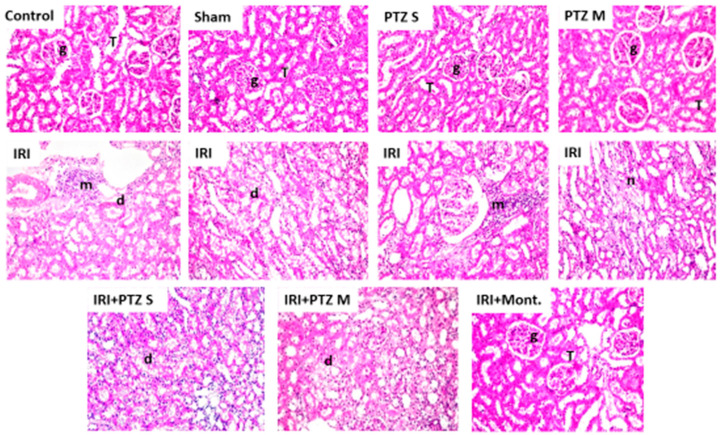
Histopathological examination of the renal tissues of different groups. Hematoxylin–eosin-stained sections of kidneys of the control-group rats (Control), sham-group rats (Sham), PTZ S-group rats (PTZ S), PTZ M-group rats (PTZ M), IRI-group rats (IRI), IRI + PTZ S-group rats (IRI + PTZ S), IRI + PTZ M-group rats (IRI + PTZ M), and IRI + montelukast-group rats (IRI + Mont). Where (g): the cortex glomeruli, (T): tubules, (m): periglomerular focal inflammatory cells aggregation, (d): degenerated tubules, and (n): focal coagulative necrosis. Magnification: ×40.

Histopathological examination for the renal tissue sections of the treated rats was performed to confirm the effect of pantoprazole, using montelukast as a control treatment. Groups I (normal control), II (sham), III (PTZ S), and IV (PTZ M) showed no histopathological changes, where the standard histological structure of the cortex glomeruli, tubules, and the corticomedullary and medullary section tubules were found. However, in group V (IRI), severe infiltration of focal inflammatory cells was observed in the cortex between the degenerated tubules. In addition, the corticomedullary portion also showed degeneration in the lining tubular epithelium and there was periglomerular focal inflammatory cells aggregation at the cortex in the lumen of most of these tubules. Furthermore, focal coagulative necrosis was detected in the tubules’ lining epithelium at the corticomedullary portion. Meanwhile, group VI (IRI + PTZ S) showed a less degenerative change in the lining epithelial cells of the tubules at the cortex. Moreover, group VII (IRI + PTZ M) showed unremarkable degenerative change in the tubules’ lining epithelium at the cortex. Finally, group VIII (IRI + montelukast) displayed no histopathological change. The typical histological structure of the glomeruli and tubules in the cortex and the tubules in the corticomedullary and medullary portions was reported, as shown in Figure 5.

## 3. Discussion

Renal ischemia is the most common cause of acute renal dysfunction, leading to functional damage by combining renal vasoconstriction, tubular obstruction, tubular glomerular filtrate back-leakage, and diminished glomerular permeability [30]. IRI contributes to the stimulation of different cell-damage mechanisms, including the formation of ROS [31,32].

Screening for novel applications for natural [19,33] or synthetic [34,35,36] candidates, modulating various pathways, became a remarkable approach [37,38]. In the current study, we showed that IRI increased the lipid peroxidation by-product MDA and increased Scr and BUN levels, as previously reported [39,40,41]. By contrast, rats treated with pantoprazole against acute kidney injury showed a reduction in MDA levels, a decrease in Scr and BUN levels, and improvement in the renal dysfunction following IRI.

Our results showed that PTZ modulated IRI by acting as an ROS scavenger, resulting in reduction in renal lipid peroxidation. It guarded against IRI by reducing the inflammatory response. Its administration decreased serum levels of pro-inflammatory cytokines, including IL-1β, TNF-α, and IL-6, and attenuated MAPK (ERK1/2, JNK1/2, p38)–NF-κB, and apoptosis signaling pathways after IRI in rats.

NF-κB is an essential transcription factor that binds to DNA regulatory sequences in cells and regulates the rate of gene expression of pro-inflammatory mediators. Once triggered (following IRI), NF-κB protein is up-regulated to control the transcription of target genes, which are responsible for the expression of various inflammatory cytokines (TNF-α, IL-6, IL-1β) [42]. After the activation of macrophages, the MAPK pathway, including ERK1/2, JNK1/2, and p38, is activated, which is an essential factor in controlling apoptosis and the release of pro-inflammatory cytokines [43]. The activation of the MAPK signaling pathway was closely associated with NF-κB up-regulation [44,45]. Our results showed that PTZ decreased cytokines production by down-regulation of NF-κB protein and by inhibiting the phosphorylation of p38, JNK1/2, and ERK1/2 proteins, resulting in modulation of the MAPK (ERK1/2, JNK1/2, p38) signaling pathway to attenuate ischemia-induced inflammatory injury and apoptosis.

The exact relationship between mitochondrial ROS and pro-inflammatory cytokines, such as IL-1β, TNF-α, and IL-6 which affect anti-apoptotic Bcl-2 and pro-apoptotic *Bax* protein expression, is still unclear. The pattern of expression of these proteins and their relationship are related to the toxicity of pro-inflammatory cytokines and their dependence on antioxidant defense status [23,46,47]. It has been assumed that the direct harmful effects of mitochondrial ROS, including cardiolipin peroxidation, facilitate the transition to mitochondrial permeability, and inhibit mitochondrial metabolism. These direct toxic effects of mitochondrial ROS, and the impacts of Mito Catalase overexpression on the *Bax/Bcl-2* relative gene expression, indicate the crosstalk between the mitochondrial ROS generation and the *Bax/-Bcl-2* expression [48]. It was reported that the activation of MAPK and up-regulation of NF-κB have contributed to the up-regulation of *Bax* expression and down-regulation in *Bcl-2* expression, resulting in apoptosis [49]. Consequently, the increased expression of *Bax/Bcl-2* exerts a strong apoptosis-promoting capacity, leading to changes in the mitochondrial membrane potential and structure, and then triggering the apoptosis pathway [50]. We demonstrated that the pro-apoptotic *Bax* gene is dramatically expressed at higher levels compared to the anti-apoptotic *Bcl-2* gene in IRI rats. After treatment with PTZ, the pro-apoptotic *Bax* gene was significantly down-regulated and the expression of anti-apoptotic *Bcl-2* gene was markedly up-regulated. To confirm apoptosis, the expression of *Bax/Bcl-2* needs to be monitored by caspase-3, which is one of the major effectors of apoptosis, and its activation indicates cell apoptosis. The activation of the apoptotic cascade results in cleavage of procaspase. Therefore, this cleaved caspase-3 is a good indicator for apoptosis [51,52]. We showed that the cleaved caspase-3/caspase-3 proteins ratio was elevated in IRI rats and reduced after treatment with PTZ. Taken together, we demonstrated that PTZ protected against renal IRI and inhibited apoptosis by down-regulating the expression of *Bax/Bcl-2* genes and cleaved caspase-3/caspase-3 proteins ratio.

## 4. Materials and Methods

### 4.1. Drugs and Chemicals

All chemicals were of analytical grade and were secured from commercial sources. Pantoprazole sodium (98%, Taketa GmbH company, Byk-Gulden-Str. 2, Germany) was prepared in 0.9% Sodium Chloride Injection solution [53] at concentration of 40 mg/mL and stored in dark at 4 °C. Montelukast (Sedico Pharmaceutical Company, Egypt) powder was freshly prepared at 10 mg/mL in 0.9% Sodium Chloride Injection solution.

### 4.2. Animals and Care

Male Wistar albino rats (*n* = 64, 255–300 g, 6–9 weeks old) were housed in cages with free access to water and food. The research protocols and animal care were carried out in compliance with the rules established by the Research Ethics Committee, Minia University, Egypt (A2020-PH-12).

Renal IRI was conducted as follows. Rats were anesthetized with xylazine hydrochloride (10 mg/kg intraperitoneal (I.P.) and ketamine (50 mg/kg (I.P.)) [54]. Bilateral renal pedicle occlusion was performed with atraumatic microvascular clamps for 45 min. At the end of the ischemic period, the clips were removed to enable blood reperfusion. After the clamps were removed, the kidneys were examined for 1 min to preserve blood supply, as shown by a return to their original color, and then the abdomen was closed with a moist sterile pad and surgical forceps. Sham-operated rats underwent the same surgical treatments, except that atraumatic microvascular clamps were not implemented. To shield rats from hypothermia, the operating table was heated with a heating pad during the study, and the rectal body temperature was measured with a probe and held at 37–37.5 °C. To resist dehydration, 3 mL/kg/h of subcutaneous 0.9% of the warm saline fluid solution and nalbuphine (0.0045 mg/100 g of body weight) were administered postoperatively. This was repeated as necessary every 12 h.

### 4.3. Experimental Design

Rats were divided into eight groups. Each group contained eight rats (*n* = 8).

Group I (normal control): rats received saline (0.2 mL) with (I.P.) injection.

Group II (sham group): rats were sham-operated by exposing the renal arteries and then closing the incision without inducing IRI and received saline (0.2 mL) with (I.P.) injection.

Group III (PTZ S): rats were sham-operated as described above and received a single dose of PTZ (160 mg/kg) by (I.P.) injection [55].

Group IV (PTZ M): rats were sham-operated as described above and received multiple doses of PTZ (40 mg/kg, orally) twice daily for ten days [55].

Group V (IRI): rats were subjected to 45 min of renal pedicle occlusion followed by reperfusion and received saline (0.2 mL) with (I.P.) injection for ten days.

Group VI (IRI + PTZ S): rats were IRI operated as described above and received a single dose of PTZ (160 mg/kg) by (I.P.) injection [55].

Group VII (IRI + PTZ M): rats were IRI operated as described above and received multiple doses of PTZ (40 mg/kg, orally) twice daily for ten days [55].

Group VIII (IRI + montelukast) as a positive control group: rats were IRI operated as described above and received a single dose of montelukast (10 mg/kg, I.P.) [29].

At the end of the experiment, rats were fasted for 12 h and allowed access only to water before scarification. Rats were sacrificed after 24 h or ten days according to each group. Serum was drained from cardiac blood and was stored at −20 °C for the biochemical analysis. Renal tissues were rapidly dissected out, washed with cold (1X) phosphate-buffered saline (PBS, pH 7.4, with a final concentration of 10 mM PO_4_^3−^, 137 mM NaCl, and 2.7 mM KCl) in ice, dried, and weighed. Renal tissue samples were divided into three parts. One part was stored for western blotting after homogenization in lysis buffer (20 mM Tris, 1 mM EDTA, 100 mM NaCl, protease inhibitors mix [56,57], and 0.5% Triton X-100 buffer). The second part was kept in RNA latter then stored at 4 °C for 24 h, and after that stored at −20 °C for measuring mRNA by quantitative real time-polymerase chain reaction (qRT-PCR), and finally, the third part was fixed in formalin 10% for Hematoxylin–eosin staining (H&E staining).

### 4.4. Biochemical Analysis

Renal functions were measured by estimation of Scr by an Scr-determination kit (human diagnostic, Wlesbaden, Germany #10051) and estimation of BUN levels in serum by a blood urea kit (human diagnostic, Wlesbaden, Germany #10505). Oxidative stress was estimated by MDA levels in serum using an MDA estimation kit (Biodiagnostic, Egypt # CAT. No. MD 2529). The spectrophotometric assessment of all these biochemical parameters was performed according to the manufacturer’s instructions using commercially available kits.

### 4.5. Measurement of Serum Levels of Inflammatory Cytokines

Enzyme-linked immunosorbent assay (ELISA) kits (Elabscience^®^, Houston, TX, USA) were used for the determination of serum levels of TNF-α (Catalog Number: E-EL-R001996T), IL-1β (Catalog Number: E-EL-R001296T), and IL-6 (Catalog Number: E-EL-R001596T) according to the manufacturer’s instructions.

### 4.6. Quantitative Real-Time Polymerase Chain Reaction

Approximately 100 mg of the kidney was homogenized by ultrasonic homogenizer (SFX 550 Branson Digital Sonifier^®^ ultrasonic cell disruptor/homogenizer is versatile. Danbury, CT, USA) in 1 mL of TRIzol TM reagent (Amresco, Solon, OH, USA). RNA was extracted from renal tissue using the TRIzol TM RNA Extraction Reagent (Amresco, Solon, OH, USA) as instructed by the manufacturer. The overall RNA concentration was estimated at A260 nm, and the purity was measured based on the ratio A260/A280. Samples with purity ≥ 1.7 were used for qRT-PCR. *GAPDH* was used as a reference housekeeping gene. cDNA synthesis was performed for equivalent amounts of total RNA in all samples using the RevertAid H Minus First Strand cDNA Synthesis Kit (#K1632, Thermo Science Fermentas, St. Leon-Ro, Germany) as directed by the manufacturer. Real-time PCR was conducted with single-stranded cDNAs. The sequences of the used primers are shown in Table 1. PCR reactions were conducted by SYBER Green (#K0251, Thermo Scientific Fermentas St. Leon-Ro, Germany-Maxima SYBER Green qPCR Master Mix (2X)) using a StepOne Real-Time PCR Detection System (Applied Biosystems).

Real-time polymerase chain reaction (qRT-PCR) was achieved using 20 μL of RealMOD Green qRT-PCR Mix kit (iNtRON biotechnology) with 0.02 μg RNA per reaction and10 Pmol of unique primers, for 30 cycles of 95 °C for 10 s. and 60 °C for 1 min. The comparative Ct (threshold cycle) approach was used to assess the relative concentrations of the products. The relative expression was determined using formula 2^(−ΔΔCt)^. They were scaled compared to the controls.

### 4.7. Western Blotting Analysis

Parts of the renal tissues were homogenized in T-PER protein extraction reagent (Thermo Fisher Scientific Life Sciences, Waltham, MA, USA) using an ultrasonic homogenizer (SFX 550 Branson Digital Sonifier^®^ ultrasonic cell disruptor/homogenizer is versatile. Danbury, CT, USA). The homogenates were centrifuged, and the protein concentrations of the supernatants were estimated, using a protein assay kit (Bio-Rad Laboratories Inc., Hercules, CA, USA). A total protein of 50 µg of each homogenate was transferred to a polyvinylidene difluoride (PVDF) membrane (EMD Millipore, Billerica, MA, USA), after electrophoresis on a 12.5% sodium dodecyl sulfate-polyacrylamide (SDS-PAGE) gel. Non-specific membrane-binding sites were blocked with 5 percent non-fat dry milk for 1 h at room temperature. The membranes were then incubated with specific primary antibodies at 4 °C overnight. Proteins: p-38 (1:1000, Cell Signaling Technology, #8690), phospho-p-38 (1:1000, Cell Signaling Technology, #4511), phosphor-ERK (1:1000, Cell Signaling Technology #9102), ERK (1:1000, Cell Signaling Technology #4370), NF-κB p65 (1:5000, Abcam, Ab32536), JNK (1:1000, Cell Signaling Technology, #4672), p-JNK (1:100, Cell Signaling Technology, #9251), cleaved caspase-3 (1:1000, Cell Signaling Technology #9661), caspase-3 (1:1000, Cell Signaling Technology #9662), and β-actin (sc-1615, 1:250) (Santa Cruz Biotechnology, Santa Cruz, CA, USA). Three washes for the PVDF membrane with washing buffer (PBS, 0.1%Tween 20, and 0.1 percent BSA) eliminated the excess of primary antibody. After washing, the membranes were further incubated for 1 h with the corresponding horseradish peroxidase-conjugated secondary antibodies at room temperature. The protein bands were visualized by chemiluminescence using the ECL detection system (Amersham Bioscience, Freiburg, Germany). The band amplitude was quantified to β-actin bands by densitometry with the Gel-Pro Analyzer 6.0 software (Media Cybernetics, Silver Spring, MD, USA).

### 4.8. Histological Examination

Autopsy samples were collected from kidneys in various groups and embedded at 10% formalin saline for 24 h. Washing was performed in tap water, and serial alcohol dilutions 10–100% (ethyl, and absolute ethyl) were used for dehydration. Specimens were cleared in xylene and put in paraffin at 56 °C in the hot air furnace for 24 h. Paraffin bees-wax tissue blocks were prepared for sectioning by slide microtome at 4 microns thickness. The tissue parts obtained were collected on glass slides, deparaffinized, and stained with (H&E) stain for routine inspections by a light electrical microscope (Olympus BH 2, Tokyo, Japan) [58].

### 4.9. Statistical Analysis

The data were encoded and entered using the Graph Pad Prism version 7 statistical package (GraphPad, La Jolla, CA, USA). Statistical variations between groups were evaluated by Student’s *t*-test after one-way analysis of variance (ANOVA). *p*-values smaller than or equal to 0.05 were deemed to be statistically significant.

## 5. Conclusions

This research demonstrated, for the first time, that the potent anti-inflammatory and anti-apoptotic effects of pantoprazole were responsible for the attenuation of IRI that induced renal injury and apoptosis. It suppressed apoptosis via modulation of the expression of *Bax* and *Bcl*-2 genes and cleaved caspase-3 protein. Furthermore, it attenuated the extracellular signaling pathways, the pro-inflammatory cytokines (TNF-α, IL-1β, and IL-6), and deactivated the intracellular signaling pathways, MAPK (ERK1/2, JNK, p38), and NF-κB.

## Figures and Tables

**Table 1:** Primers sequences used in qRT-PCR.

Primer	Sequence of the Primer
*BAX*	Forward: 5′-GGT GTT GAC GGT TCA CTT GC-3′ Reverse: 5′-AAC GCC TGG ATG GGC TTT TA-′.
*Bcl-2*	Forward: 5′-TGT ATC AAA CCA TGC GGC TG-3′ Reverse: 5′-GGC TGG TTT TAC CGC ACC TT-3′.
*GAPDH*	Forward: 5′-ACC AAC TGC TTA GCC CCC C-3′ Reverse: 5′-GCA TGT CAG ATC CAC AAC GG-3′.

## Data Availability

All data are fully available and included in the manuscript except replicates of blots which are not available in the manuscript.
